# Red blood cells could protect miRNAs from degradation or loss thanks to Argonaute 2 binding

**DOI:** 10.1002/2211-5463.70005

**Published:** 2025-04-15

**Authors:** Elena Perla, Faiza Abbas, Luigia Rossi, Mauro Magnani, Sara Biagiotti

**Affiliations:** ^1^ Department of Biomolecular Sciences University of Urbino Italy

**Keywords:** Ago2, circulating miRNAs, drug delivery system, microRNAs, minimal RISC, Red blood cells

## Abstract

Red blood cells (RBCs) have emerged as reservoirs of microRNAs (miRNAs) in the circulatory system, challenging the traditional view of their nucleic acid absence. This study investigates the miRNA profiles and stability of both native and engineered RBCs. We demonstrate that RBCs are rich in miRNAs, which remain stable under physiological conditions, likely due to their association with Ago2, a key RNA‐binding protein. The stability and retention of miRNAs persist even after hypotonic dialysis used for RBC engineering. These findings underline the potential of RBCs as miRNA carriers for therapeutic applications and as a foundation for RNA‐based delivery systems. Such advancements could redefine their role in transfusion medicine and advanced RNA therapies.

## Graphical abstract

The present work investigates the presence and the stability of miRNAs into RBCs, both native and engineered. The observed stability is due to the Ago2 bound resulting in minimal RISC. Indeed, if the miRNA‐Ago2 complex is present, miRNAs are protected from release or degradation and they are biologically active. Thus, RBCs can act as miRNA carriers for RNA‐based delivery systems.

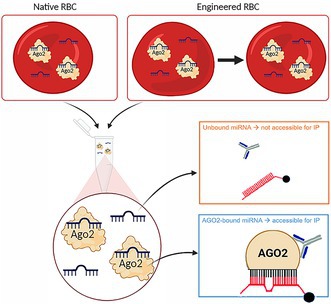

**Abbreviations**
Ab: antibody
Ago2: Argonaute2
EVs: extracellular vesicles
IP: immunoprecipitation
miRNAs: microRNAs
ND: not dialysed
PLB: polysome lysis buffer
qPCR: quantitative polymerase chain reaction
RBCEVs: red blood cells‐derived extracellular vesicles
RBCs: red blood cells
RIP: RNA immunoprecipitation
RISC: RNA I silencing complex
snRNA: small nuclear RNA
UB: unbound
UL: un‐loaded
WB: whole blood
WBC: white blood cells

Human red blood cells (RBCs) are terminally differentiated, enucleate cells particularly rich in haemoglobin and primarily involved in gas transport and exchanges [1]. Despite RBCs being long thought to lack nucleic acids, a recent comprehensive joint analysis of the long and short RNA transcriptomes of human RBCs revealed an extensive repertoire of mRNAs that encode many critical proteins for RBC differentiation and function [2]. Up to now, 359 microRNAs (miRNAs) have been identified in mature RBCs, and multiple miRNAs seem highly expressed. The top 10 miRNAs with the highest expression include miR‐451a, miR‐144‐3p, miR‐16‐5p, miR‐92a, let‐7 and miR‐486‐5p [2]. In the last years, researchers hypothesized that RBCs could be potential repositories of miRNAs in the circulatory system and, under specific stimuli, could release extracellular vesicles (EVs) containing miRNAs to target recipient cells [2, 3]. RBCs could provide miRNAs to recipient cells in two different ways. The large circulating RBCs may be the source of miRNAs associated with proteins; protein‐bound miRNAs are transported to recipient cells where these reprogram mRNA translation [3]. However, extracellular vesicles‐mediated miRNA transmission is the main pathway for RBC miRNA function. Under normal physiological conditions, RBC‐derived EVs constitute 7.3% of EVs in whole blood, indicating that RBCs are one of the primary sources of EVs in peripheral blood [4, 5]. In addition, Juzenas and collaborators showed that 38 miRNA species are shared by serum, exosomes and RBCs, suggesting that RBCs may be the main source of both [6]. Moreover, Huang's group has detected that 78 miRNAs are present in exosomes of stored RBCs, of which miR‐125b‐5p, miR4454 and miR‐451a result in the most abundant [7].

Notably, miRNAs are ‘small noncoding’ RNAs (18–25 nucleotides) that negatively regulate gene expression, yet they are involved in several physiological and pathological functions, including cell proliferation, cell differentiation, apoptosis, angiogenesis, tumour development and haemopoiesis [8, 9, 10]. miRNAs can also bind to proteins, mainly Argonaute (Ago). Ago represents a highly conserved family of proteins that acts as a key player in RNA silencing, constituting the catalytic core of the RNA‐induced silencing complexes (RISC) for controlling protein synthesis and RNA stability [11]. Attention has been paid to RBC miRNAs in transfusion medicine. Indeed, it has been shown that stored RBCs undergo the so‐called ‘storage lesions’ involving morphological, physiological and biochemical changes, where miRNAs have important functions in cell apoptosis and life processes [12, 13, 14]. For example, Chen et al. demonstrated that eight predicted miRNAs with modified expressions were dysregulated after 20‐day storage [15]. In particular, miR‐31‐5p, miR‐196a‐5p, miR‐203a, miR‐654‐3p and miR‐769‐3p were involved in apoptosis and senescence signalling pathways. Moreover, their expression was increased, whereas miR‐96‐5p, miR‐150‐5p and miR197‐3p levels were decreased. Also other authors turned their attention to this field investigating the miRNA expression levels in different storage conditions [16]. Thus, dysregulated miRNAs might represent potential biomarkers for identifying storage lesions, and their detection might help evaluate the quality of stored RBCs.

In addition, in the past decades, RBCs have been extensively explored as drug delivery systems due to their biocompatibility, long circulation time and natural ability to transport substances within the bloodstream. The concept emerged in the 1980s, when techniques to encapsulate therapeutic agents, such as enzymes and drugs, into RBCs were developed, demonstrating their potential as carriers for sustained drug release [1, 17, 18, 19] Advancements in RBC engineering have enabled the delivery of large molecules and targeted therapies, with techniques like surface modification and nanoencapsulation enhancing their use in precision medicine [20, 21, 22]. These carriers continue to evolve, offering promising solutions for disease treatment while reducing side effects. Thus, several RBC‐inspired strategies have been developed, including native RBCs, ghosts, RBC‐mimetic nanoparticles and RBC‐derived extracellular vesicles (RBCEVs) [23, 24]. Particularly, native RBCs have been used as drug delivery systems for a wide range of drugs [1, 25, 26, 27, 28, 29, 30, 31], and some applications reached the clinic owing to a patented loading method and equipment [32]. On the contrary, RBCs were also exploited as drug delivery system when engineered with the encapsulation of different molecules [33, 34, 35, 36, 37, 38, 39, 40]. The authors recently performed an in‐depth analysis of loaded RBCs' proteome and metabolome [41]. To the best of our knowledge, studies regarding the RNA content of drug‐loaded RBCs are still lacking.

The present study focused on three miRNAs naturally present in mature RBCs: miR‐106b‐5p, which is usually used as a reference gene [42]; miR‐196a‐5p, which has been associated with storage lesions [15, 42, 43]; miR‐451a the most studied RBC miRNAs being highly expressed and for its unique way of maturation [44] and exploited as a promising cancer biomarker; miR‐16‐5p for its very high expression in RBCs [2, 3]; and miR‐148a‐3p because of its downregulation in RBCs in cancer patients [45]. First, we compared the basal level of those miRNAs in a cohort of different blood donors, and then, we tried to assess their stability under different conditions. Finally, their ability to bind Ago2 has been evaluated, and their correlation to several features observed in RBC was subjected to various experimental conditions. We concluded that RBCs are rich in miRNAs, and those miRNAs have a very long stability maybe thanks to their binding to Ago2 proteins. This can represent a good starting point for building a new RBC‐based platform for short RNA delivery.

## Materials and methods

### Native RBC purification and RNA extraction

RBCs were purified from 4 to 5 mL of whole blood (WB) collected in EDTA and withdrawn from healthy donors afferent to the Transfusion Center of the Presidio Ospedaliero Unico ‘Santa Maria della Misericordia’ of Urbino, Italy, and included in the Italian blood donor registry (A.V.I.S., Associazione Volontari Italiani Sangue), who signed an informed consent form, on the basis of an official agreement document with the above mentioned Transfusion Center. The investigations were carried out after obtaining appropriate institutional review board approval following the guidelines and regulations of the Declaration of Helsinki (Determina Direttore Generale n. 1511). WB samples underwent three serial centrifugations at 1800 ***g*** for 10 min at 4 °C and between two washes in HEPES solution (HEPES 10 mm, NaCl154 mm, glucose 5 mm, pH 7.4, 300 mOsm) with 300 mOsm. Isolated RBCs were additionally leukodepleted before analyses using a leukocyte depletion filter (Acrodisc™ WBC syringe filter, Pall Life Sciences, New York, USA). The latter are the most represented contaminants of blood products, and their removal complies with the blood product processing guidelines [46, 47]. For this reason, the purity of isolated RBCs has been evaluated and reported in Table S1 and Fig. S1. A total of 100 μL RBC pellets, containing about 3–6 × 10^8^ cells in each sample, were tested. All the procedures were conducted under sterility and RNase‐free conditions. Total RNA was extracted from RBC samples using a lysis reagent and a miRNA isolation kit (Qiazol and miRNeasy mini kit, respectively, Qiagen, Hilden, Germany) according to the manufacturer's protocol. RNA quality and concentration were evaluated using the spectrophotometer NanoDrop ND‐1000 (Nanodrop Technologies, Wilmington, DE, USA). This kit enabled the extraction of both long and short RNAs with high concentration and quality indexes; indeed, RNA mean concentration was 71.82 ± 18.44 ng·μL^−1^, while mean 260/280 was 1.90 ± 0.10 and mean 260/230 1.13 ± 0.14.

For short‐term stability studies, RBCs have been resuspended at 0.5% in RPMI and incubated for different times (0, 2, 4, 8, 24 and 48 h) at 37 °C in a humidified incubator. At the end of each incubation time, samples were collected and subjected to totRNA extraction as above described.

### Production of engineered RBCs

Purified fresh RBCs underwent a loading procedure without adding any cargoes, producing ‘un‐loaded’ (UL) RBCs essentially as described in [48]. This procedure is based on hypotonic dialysis followed by isotonic resealing and reannealing of the membranes. Initially, purified RBCs were diluted in Hepes buffer at 70% and dialysed against a hypotonic buffer (NaH_2_PO_4_ 10 mm, NaHCO_3_ 10 mm, glucose 20 mm, pH 7.4, 60 mOsm) for 60–90 min at 4 °C. Then, membrane resealing was performed by the addition of a diluted hypertonic solution (inosine 100 mm, ATP 20 mm, glucose 10 mm, sodium pyruvate 100 mm, MgCl_2_ 4 mm, NaCl 190 mm, KCl 1666 mm, NaH_2_PO_4_ 33 mm), whereas an incubation at 37 °C for 25 min enabled the membrane reannealing. Lastly, the eventual not re‐entrapped RBC components were eliminated by two washes in Hepes buffer and following centrifugations at 500 ***g*** at 4 °C for 10 min.

### Reverse transcription and qPCR amplification

Relative changes in miRNA abundance were quantified using Taqman Small RNA assays (Applied Biosystems, Waltham, MA, USA) using the following probes: hsa‐miR‐106b‐5p, hsa‐miR‐196a‐5p, hsa‐miR‐451a, hsa‐miR‐16‐5p and hsa‐miR‐148a‐3p (Table S2). These include a miRNA reverse transcription step to create a miRNA‐specific cDNA that also introduces extension sequences that can be used as the template for qPCR in the second step using the TaqMan MicroRNA Reverse Transcription Kit (Applied Biosystems). After RNA quantification, cDNA conversion was performed from 10 ng of total RNA, according to a standard protocol. The mixture was incubated at 16 °C for 30 min to allow RT primer annealing and then at 42 °C for 30 min to let the RT enzyme work. Reactions were stopped by incubating them at 85 °C for 5 min and finally cooled to 4 °C. Samples were used fresh or stored at −20 °C. Briefly, 1 μL cDNA was used for quantification by qRT‐PCR. Subsequent qPCR was performed using an ABI 7500 instrument in either duplicate or triplicate with a 96‐well block and reaction volumes of 20 μL. Each reaction consisted of 1 μL of the diluted reverse transcription reaction, 10 μL of 2× Universal PCR Master Mix, No AmpErase UNG (Applied Biosystems), 1 μL of the specific 20× Taqman Assay and 8 μL nuclease‐free water. The reaction conditions consisted of polymerase activation or denaturation and well‐factor determination at 95 °C for 10 min, followed by 40 amplification cycles at 95 °C for 10 s and 60 °C for 1 min. CT values were determined using an automated baseline and threshold, and the mean CT was determined from the triplicate PCR results. The relative expression of each gene was calculated using the comparative CT (2−^▵▵CT^) method. miR‐106b‐5p or U6 snRNA was used as an internal control, and its expression stability was tested in RBCs.

### Absolute quantitation of miRNAs

Absolute quantification was made based on [49] with minor modifications. PCR‐based absolute quantification involves the amplification of standards at known concentrations to form a standard curve based on Ct values. We, therefore, used synthetic miRNAs (5′ phosphorylated RNA oligonucleotides, Merck) as the template for miRNA reverse transcription reactions. These corresponded to the miRbase sequences of hsa‐miR‐106b‐5p, hsa‐miR‐196a‐5p, hsa‐miR‐451a, hsa‐miR‐16‐5p and hsa‐miR‐148a‐3p (Table S2). The oligonucleotides were resuspended in nuclease‐free water at a concentration of 100 pmol·μL^−1^ (100 pm) and stored at −80 °C. To generate the standard curve, serial dilutions of the synthetic miRNAs were reverse transcribed using the corresponding RT Primer and an input range of 0.001–100 fmol·reaction^−1^ (in 5 μL, giving a total volume of 15 μL). This was achieved by diluting each synthetic miRNA to 20 fmol·μL^−1^ (0.2 μm) and then performing six 1 in 10 dilutions in nuclease‐free water. Due to the extremely low concentration of miRNAs resulting from the experiment, dilutions were made from fresh stocks on each occasion. Test samples were reverse transcribed in parallel with the standard curve. These were either 10 ng total RNA or 5 μL of the standard dilutions. Subsequently, all standards and test samples were handled identically, and absolute quantitation was performed on the ABI 7500HT with the same reaction setup as described above. A representative standard curve obtained in this way is reported in Fig. S2. This analysis gave results with the units ‘fmol’. The cellular abundance of a particular miRNA was expressed as fmoles per nanogram of total RNA.

### RNA immunoprecipitation

To investigate Ago2‐miRNA complexes, an RNA immunoprecipitation (RIP) protocol was set up. Actually, several protocols and conditions were tested (for a detailed report, see Table S3). The final optimized protocol was set up based on [50] with some modifications. Briefly, 100 μL RBC sample was immunoprecipitated in 1 volume (v/v) of polysome lysis buffer (PLB) added with 1 mm DTT, 200 U·mL^−1^ RNase OUT and 1× Complete Mini EDTA‐free protease inhibitor. 75‐μl Dynabeads protein G (Thermo Fisher) were washed twice and resuspended in NT‐2 buffer; 5 μg control IgG or anti‐Ago2 antibodies were then added and let incubate for 1 h at 4 °C according to the preincubation protocol. Ab‐beads complexes were finally washed and used to capture Ago2‐miRNA complexes from RBC native lysates. Refer to [30] and/or Table S3 for reagents and more detailed RIP conditions [50]. Notably, EDTA was not added to the NT‐2 buffer during the capture. IP and control samples were first analysed by western blotting to check Ago2 protein immunoprecipitation. For RNA isolation from the Ago2 IP sample, 700 μL of lysis reagent (Qiazol) was added directly to AGO2‐Dynabeads. totRNA extraction was carried out with miRNeasy kit (Qiagen) following the manufacturer's extraction protocol. RNA samples were used fresh or stored at −80 °C. 1 to 5 μL of each RNA sample were used for cDNA synthesis and qPCR amplification under the same conditions mentioned before for the Taqman Small RNA Assay.

### Statistical analysis

Statistical analyses were performed with GraphPad Prism 6 software using the one‐way ANOVA to compare whether two or more samples' means are significantly different. Multiple comparisons tests were used to determine whether the mean difference between groups is statistically significant.

## Results

**Fig. 1:**
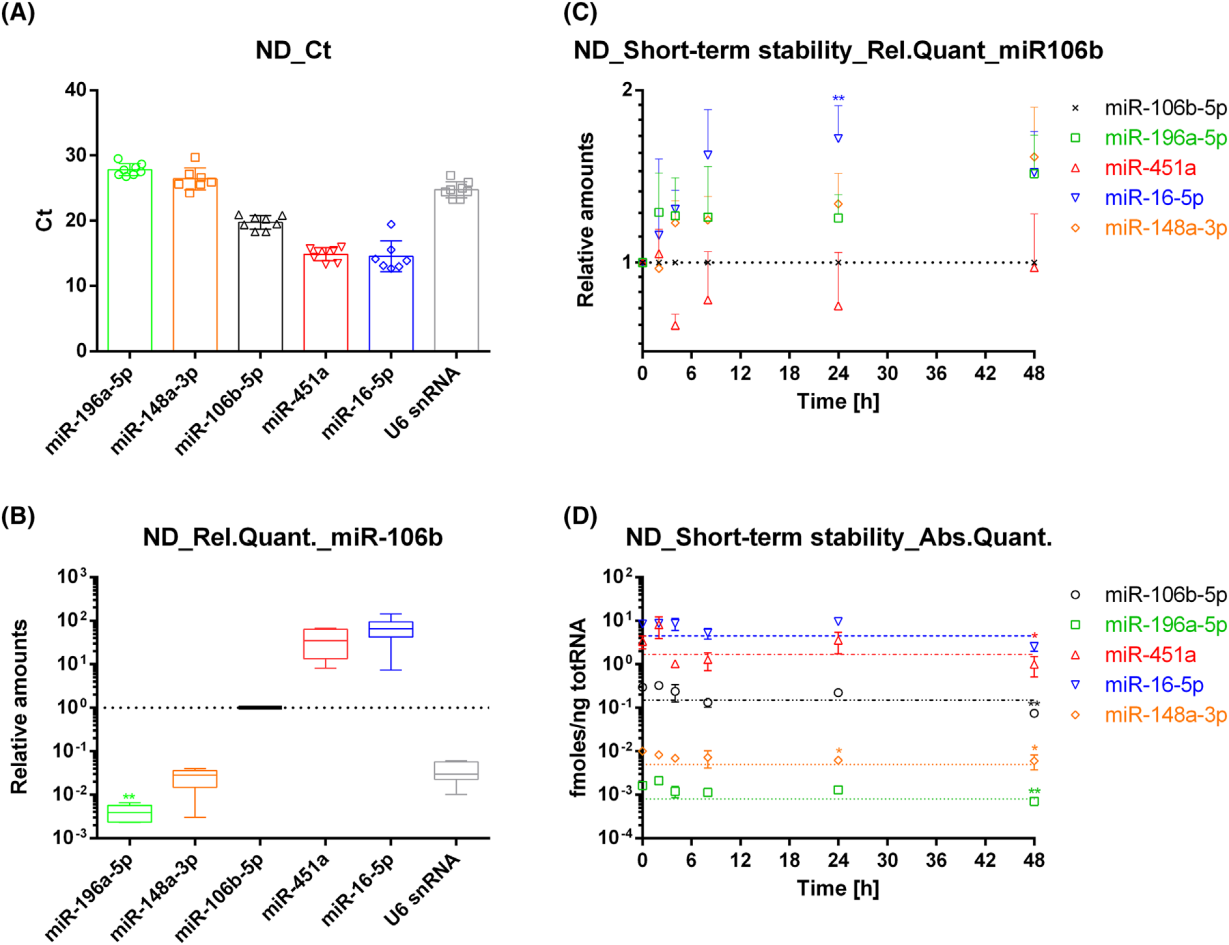
miRNA amount and stability in native red blood cells (RBCs). (A) Mean Cts of, miR‐196a‐5p, miR‐148a‐3p, miR‐106b‐5p, miR‐451a, miR‐16‐5p and U6 snRNA with the respective SEM. RT‐PCR was performed on 1 μL of cDNA and Cts values were obtained by using automated baseline and threshold. Each reaction was made in triplicate to allow the mean calculation. (B) Relative quantification of miRNA amount with miR‐106b‐5p as reference gene by using the comparative Ct method with the respective SEM (*n* = 8 in Panels A, B). (C) Short‐term stability studies by comparative dCt method using miR‐106b‐5p as reference gene with the respective SEM. In this case, RBCs were incubated at 37 °C for different time points (0–48 h). (D) Short‐term stability studies by absolute quantification the respective SEM (as reported in Fig. S3) (*n* = 4 in Panels (C, D). *P*‐values * < 0.05; ** < 0.01. Significance was assessed using one‐way ANOVA followed by multiple comparisons test.

Extensive profiling of the RBC transcriptome is critical for understanding erythrocyte biology, and recently, it has been shown that the RNA composition of erythrocytes may also change during long‐term storage for future blood transfusion [2]. In addition, blood and especially RBCs are powerful tools for transfusion medicine and biotechnological clinical approaches [1, 24, 34, 36, 51]. In light of the above, we tried to better characterize human native RBCs' miRNA content. First, purified RBCs from fresh whole blood were collected from a cohort of healthy volunteers. Donors were indifferently male or female, with a mean age of 44.67 years, while, regarding the RBC parameters, they were all in the physiological range. In Table S4, the RBC parameters of the purified RBCs are reported. Moreover, we assessed the purity of isolated RBCs. The removal of granulocytes was confirmed by the haemocytometer analysis in Table S1 and by the expression level of a specific WBC miRNA (Fig. S1). Then, we focused on five miRNAs (miR‐451a, miR‐196a‐5p, miR‐148a‐3p, miR‐16‐5p and miR‐106b‐5p) that are, respectively, involved in erythropoiesis and storage lesions [3, 15, 42, 43, 44, 52], while the last is usually used as a reference gene in this kind of analyses [42]. Finally, U6 snRNA was also tested as a reference gene. To do this, we set up total RNA extraction, small RNA synthesis and PCR amplification conditions, which allowed us to detect all the miRNAs in all the tested samples. In Fig. 1A, the mean Cts are reported together with the SD. As reported in the literature, miR‐16‐5p showed the highest expression with a mean Ct of 13.73 ± 1.05, followed by miR‐451a with a mean Ct of 14.86 ± 0.94, while miR‐196a‐5p was the lowest one with a mean Ct of 27.52 ± 0.86. miR‐148a‐3p is the second less expressed one in RBCs with a mean Ct of 25.86 ± 0.98 The small RNAs usually used to normalize target miRNAs showed an intermediate expression with a mean Ct of 19.75 ± 0.96 and 24.75 ± 1.15 for miR‐106b‐5p and U6 snRNA, respectively. Relative amounts of the target miRNAs were then calculated using both miR‐106b‐5p and U6 snRNA as endogenous controls (Fig. 1B), showing a very irrelevant intra‐donor variability.

Afterwards, we analysed the short‐term stability of the investigated mRNAs at different time points from 0 to 48 h. Figure 1C shows the results normalized to miR‐106b‐5p. As shown, by using miR‐106b‐5p as endogenous control, we obtained an apparent increase in the miR‐196a‐5p at 8 and 48 h, as for miR‐148a‐3p. This is consistent with that reported in the literature, where the increase is related to long‐term storage and blood blank quality [15, 42, 43]. In contrast, miR‐451 showed an apparent decrease over time at all times tested, while miR‐16‐5p expression seems unstable. However, we noticed that miR‐106b‐5p was also affected by a certain grade of variability over time. Thus, we repeated the analyses choosing U6 snRNA as reference gene. Unfortunately, U6 snRNA was even less stable than miR‐106b‐5p and resulted in a very rapid decrease after 24 h‐incubation (data not shown). Thus, we moved to an absolute quantification approach. As reported in the method section, we set up standard curves using serial dilutions of synthetic miRNAs, and we used the same to calculate the absolute amount of the respective miRNA (Fig. 1D). Surprisingly, all the selected miRNAs resulted quite stable over time. In detail, none showed a decrease higher than 30% up to 24 h, and miR‐148a‐3p seemed the most stable, maintaining more than 60% of its content up to 48 h. In conclusion, absolute quantification seemed the most reliable way to assess miRNA loss and/or stability inside *ex vivo* stored/incubated RBCs.

**Fig. 2:**
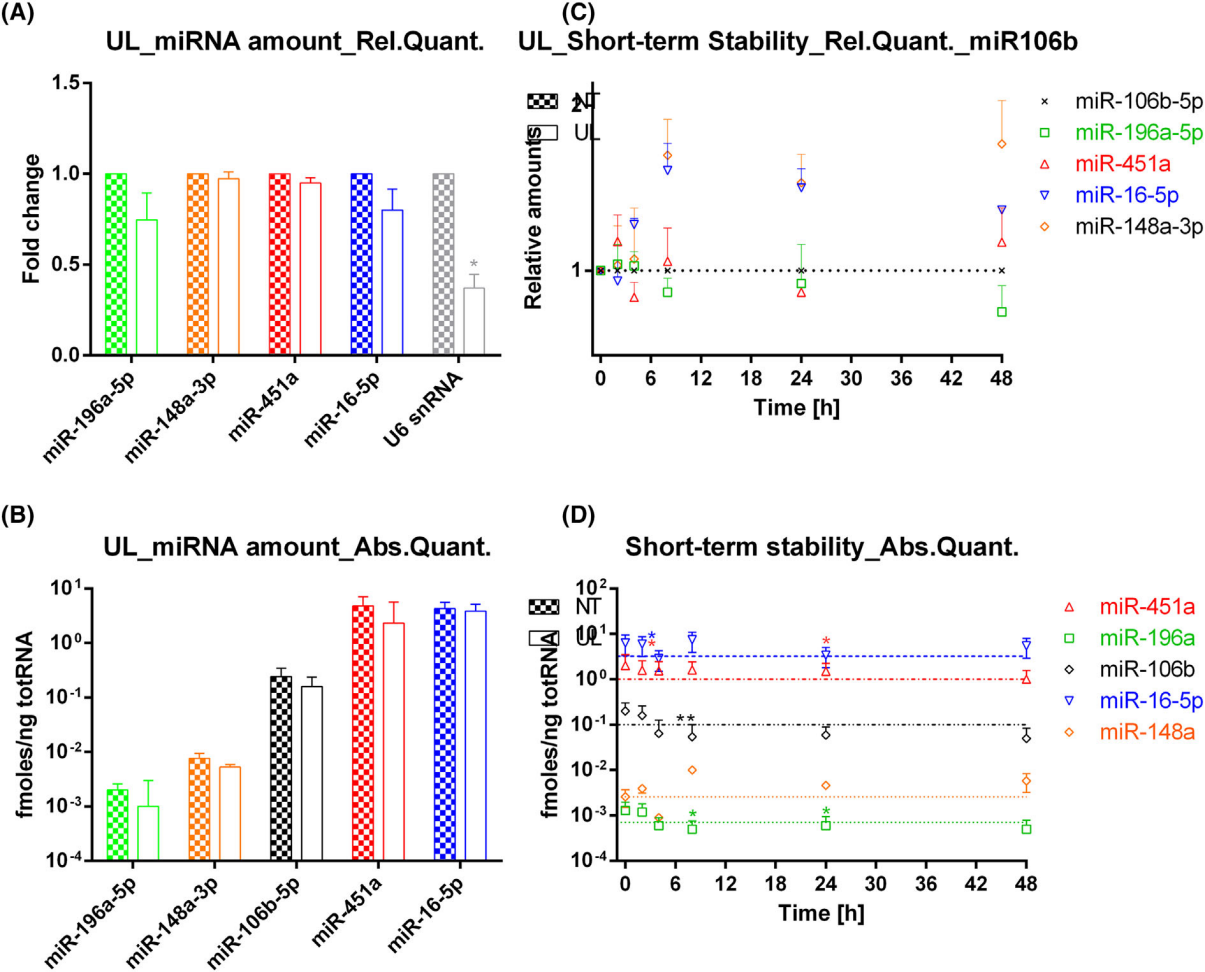
miRNA amount and stability in engineered RBCs. (A) Red blood cells (RBCs) miRNA amount in UL (un‐loaded, engineered) RBCs compared with ND (not dialysed, native) ones by relative (A) and absolute quantification (B). In A, results are expressed as fold changes with the respective SEM. In B, results are expressed as fmoles per nanogram of total RNA with the respective SEM. (C) Short‐term stability studies by comparative dCt method using miR‐106b‐5p as reference gene with the respective SEM. (D) Short‐term stability studies by absolute quantification with the respective SEM. *P*‐values * < 0.05; ** < 0.01. (*n* = 4) Significance was assessed using *t*‐test.

Then, we wanted to evaluate the amount and the stability of the selected miRNAs in ‘engineered’ RBCs that are usually produced to be used as drug delivery system. To this end, RBCs were subjected to the loading procedure without the addition of any molecules (typically, these samples are named ‘un‐loaded’ or ‘UL’ samples). Finally, we measured the amount of miRNAs before (‘not dialysed’, ND sample) and after the loading procedure (UL). We saw an initial loss of small RNAs due to the opening and resealing of the cell membrane; however, for all the miRNAs, this loss was <25% (Fig. 2A). By contrast, we lost more than 50% of U6 snRNA, which suggest a different behaviour compared with miRNAs. The insignificant miRNA loss was confirmed also by absolute quantification (Fig. 2B). After this, we repeated the same short‐term stability experiment, as before. Also in this case, we performed relative quantification using miR‐106b‐5p as a reference gene (Fig. 2C) and absolute quantification (Fig. 2D). Both showed that miRNAs are quite stable over time also in UL RBCs since 50–85% of all endogenous miRNAs tested were still inside RBCs after 48 h.

**Fig. 3:**
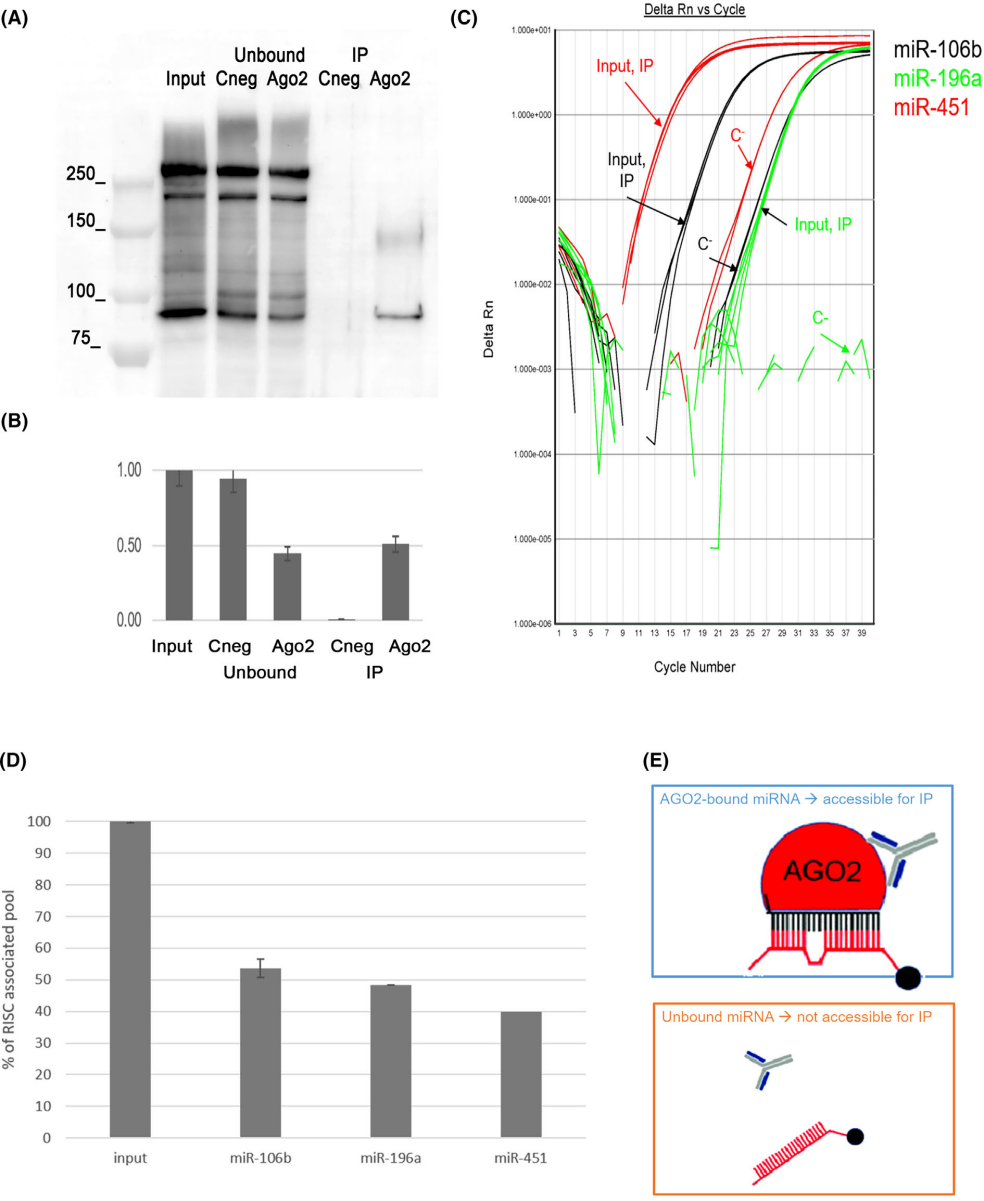
RNA immunoprecipitation (RIP) and Argonaute2 (Ago2)‐bound miRNAs. (A) Representative western blotting of input, unbound (UB) and immunoprecipitated (IP) protein samples obtained from native IP. The bands displayed at ~97 kDa are representative of the immunoprecipitation of the Ago‐2 protein in the IP samples. (B) Quantification of Ago2 bands in Input, UB and IP samples. The IP efficacy, calculated by comparing total Ago2 present in input and IP samples, is 52 ± 1%. (C) Representative amplification plots of the qRT‐PCRs performed on the different RNA samples obtained from RIP that shows the different amounts of the selected miRNAs in the three fractions input, UB and IP. IP's amplification curves are similar to the input ones, thus demonstrating that miRNAs are retained in Ago2‐IP complexes. (D) Percentage of Ago2‐bounded miRNAs compared with the unbound counterpart. These values have been normalized to the RIP efficiency (*n* = 4). (E) Outline of the principle of RIP. Recognition by the anti‐Ago2 antibody occurs exclusively for Ago2‐bound miRNAs; on the contrary, if miRNAs are free, they are not recognized by the anti‐Ago2 Ab and are not accessible for IP.

However, these findings sparked our curiosity about whether this stability could be associated with the presence of Ago2 in RBCs. Indeed, we evaluated the amount of the selected miRNAs complexed with this protein using anti‐Ago2 antibodies to pull down the minimal RISC and evaluate the percentage of bound miRNAs. Figure 3A,B shows the amount of Ago2 protein in RBCs before and after the immunoprecipitation. The protein is quite represented in RBCs and was detectable by loading about 5 μg of total proteins and also that immunoprecipitation was able to pull down more than 50% (52 ± 1%) of the protein contained in the starting sample (input). Negative controls showed no enrichment in Ago2 demonstrating the specificity of the procedure. The immunoprecipitated (IP) samples were then used for total RNA extraction and miRNA quantification. Figure 3C represents the amplification plots used showing that input and IP samples are almost similar while negative controls are clearly shifted. Respective Cts were used to calculate the percentages of miRNAs that were complexed with Ago2 (Fig. 3D). All the tested miRNAs were abundantly bound to Ago2 (range from 41 to 57%). The relatively high binding to Ago2 might therefore account for the high miRNA stability and for their almost irrelevant loss after the loading procedure.

## Discussion

RBCs are one of the most commonly studied cell types, often used as a model system to understand the general principles of molecular genetics, biochemistry, membrane biology and cell physiology. In the last decades, native RBCs have been largely employed as drug delivery systems for many classes of drugs [1, 25, 26, 32, 33] due to their peculiar characteristics, such as biocompatibility, their nonimmunogenic or cytotoxic nature and their being a precious material readily available from the patient himself [53]. Specifically, some applications have already reached clinical use thanks to a patented loading method and the Red Cell Loader equipment, as in [32]. Previous studies have already demonstrated the pharmacokinetic properties and *in vivo* kinetics of engineered RBC, highlighting low surface phosphatidylserine exposure resulting in a longer half‐life in circulation [20, 54], a very low haemolysis rate indicative of a nondamaging engineering processes, almost comparable lifespan in circulation [55] and the preservation of their original shape [56]. For a very long time, it was thought that RBCs did not contain nucleic acids; however, more recent studies have shown a high abundance of these, especially microRNA [2, 3]. Their involvement in many physiological and pathological functions explains why researchers are pointing their attention to their possible application as diagnostic biomarkers or promising therapeutic agents [2, 23, 57, 58].

Considering the potential role of RBC in transfusion medicine and biotechnological clinical strategies, in this paper, we aim to investigate the total amount of miRNAs in both native and engineered human RBCs from a cohort of donors. We focused on miR‐451, miR‐106b‐5p, miR‐196a‐5p, miR‐16‐5p and miR‐148a‐3p, which, according to previous studies, are primarily involved in storage lesions and haemopoiesis [12, 14, 15, 42, 43]. Our results confirmed that native RBCs are mostly rich in miR‐451, whereas miR‐196a‐5p is the less present one, as in the reported literature data, with miR‐148a‐3p. Most importantly, the miRNA quantity is similar in all donors tested showing a very small inter‐donor variability. Afterwards, we evaluated the selected miRNAs' short‐term stability. We noticed that relative quantification was not the appropriate method for all these quantification studies because the miRNA amount relevantly changes in dependence on the housekeeping gene used. Hence, we observed that some results showed in papers dealing with storage lesions miRNA dysregulation might vary according to the housekeeping gene used, so absolute quantification should be employed to avoid these inconsistencies.

In addition, we explored the amount of miRNAs and their short‐term stability in engineered RBCs. Also in this case, we observed a high amount of miRNAs and, compared with native ones, we found a small loss after dialysis that wasn't higher than 20% demonstrating that the loading procedure does not induce a significant loss of inner miRNAs, contrary to what one would expect. Furthermore, these miRNAs had the same short‐term stability in physiological conditions. On the one hand, this is the first paper investigating the miRNome profile of engineered RBCs, since previous work only addressed proteomic and metabolomic profiles [41]. On the other hand, this confirm that RBCs could be taken from patients in need, conveniently engineered and easily reinfused in the same patients without any notable difference with native ones. Finally, RBC miRNAs, both endogenous and exogenous, could be used to modulate cell‐to‐cell communication [48].

Lastly, more studies have demonstrated that miRNAs are usually carried within cells and extracellular vesicles in blood and/or associated with Ago2, the most represented argonaute protein in RBCs [59, 60]. Due to RBCs' abundance in blood, they are the major source of miRNAs, [3] with Ago2 constituting the main core of the RISC and binding miRNAs [44]. In this way, Ago2s are the almost exclusive carriers of miRNAs in the bloodstream, involved in multiple physiological and pathological functions, such as erythropoiesis, cell proliferation and others [60].

In the light of the above, we performed immunoprecipitation of miRNA‐Ago2 complexes. In our experimental conditions and optimized RIP protocol, we confirmed the specific abundance of Ago2 in RBCs (as previously shown in [60]) and showed that over 50% of miRNAs were Ago2‐bound, contrary to what is currently found in literature whereby this percentage is around 16% [61]. This demonstrates that Ago2‐bound miRNAs, constituting the minimal RISC, are protected from degradation and/or release, and, more importantly, this can predict their biological activity [62]. In addition, Ago2‐miRNA complexes make the latter easily capable of joining its target mRNA to regulate its genetic expression, thus making it biologically active.

In conclusion, our findings confirm that RBCs are pivotal miRNA repository in the circulation and associate their miRNA content and stability with Ago2 binding. These findings could significantly change, once again, the way to consider RBCs as ‘bags containing haemoglobin’ and/or affect their exploitation as RNA, especially miRNA, delivery system in both transfusion medicine and RBC‐based cell therapies.

## Conflict of interest

All the authors declare that the research was conducted in the absence of any commercial or financial relationships that could be construed as a potential conflict of interest.

### Peer review

The peer review history for this article is available at https://www.webofscience.com/api/gateway/wos/peer‐review/10.1002/2211‐5463.70005.

## Author contributions

EP contributed to the conceptualization, formal analysis and data interpretation, investigation, visualization, and writing—original draft. FA contributed to the investigation. LR contributed to the supervision. MM contributed to the project administration, supervision, writing—review and editing. SB contributed to the funding acquisition, methodology, project administration, investigation, and writing—review and editing.

## Supporting information


**Fig. S1.** miR‐29b‐3p expression in RBCs and WBCs.
**Fig. S2.** Standard curve used for Absolute quantification.
**Table S1.** Red Blood Cells purification from granulocytes.
**Table S2.** Small RNA sequences and Assays used for miRNAs relative and absolute quantification by qPCR.
**Table S3.** RIP protocol optimization.
**Table S4.** RBC parameters of the blood donor court.

## Data Availability

The data that support the findings of this study are available within the article and/or the supplementary material of this article.
