# Supplementary material for: Red blood cells could protect miRNAs from degradation or loss thanks to Argonaute 2 binding
Source: FEBS Open Bio. 2025 Apr 15;15(5):810–21. doi: 10.1002/2211-5463.70005 (PMC12051026; doi:10.1002/2211-5463.70005)
Supplement: Supplementary file 1 — Fig. S1. miR‐29b‐3p expression in RBCs and WBCs. Fig. S2. Standard curve used for Absolute quantification. Table S1. Red Blood Cells purification from granulocytes. Table S2. Small RNA sequences and Assays used for miRNAs relative and absolute quantification by qPCR. Table S3. RIP protocol optimization. Table S4. RBC parameters of the blood donor court. [file FEB4-15-810-s001.docx]

# SUPPORTING INFORMATION

## Table S1 – Red Blood Cells purification from granulocytes

Fresh whole blood (WB) collected in EDTA has been analysed through the haemocytometer ABX Micros ES 60 (Horiba Medical). After the purification procedure, both RBCs and white blood cells (WBCs) were analysed in the same way.

| **Sample** | **WBC (10^3^/mm^3^)** | **RBC (10^6^)/mm^3^** | **Platelet (10^3^)/mm^3^** |
| --- | --- | --- | --- |
| 1. WB | 5.33±0.32 | 4.58±0.66 | 219±45 |
| 2. RBC | 0.01±0.04 | 23.70±3.14 | 6.33±1.9 |
| 3. WBC | 63.6±3.15 | 0.11±0.006 | 51±2.56 |


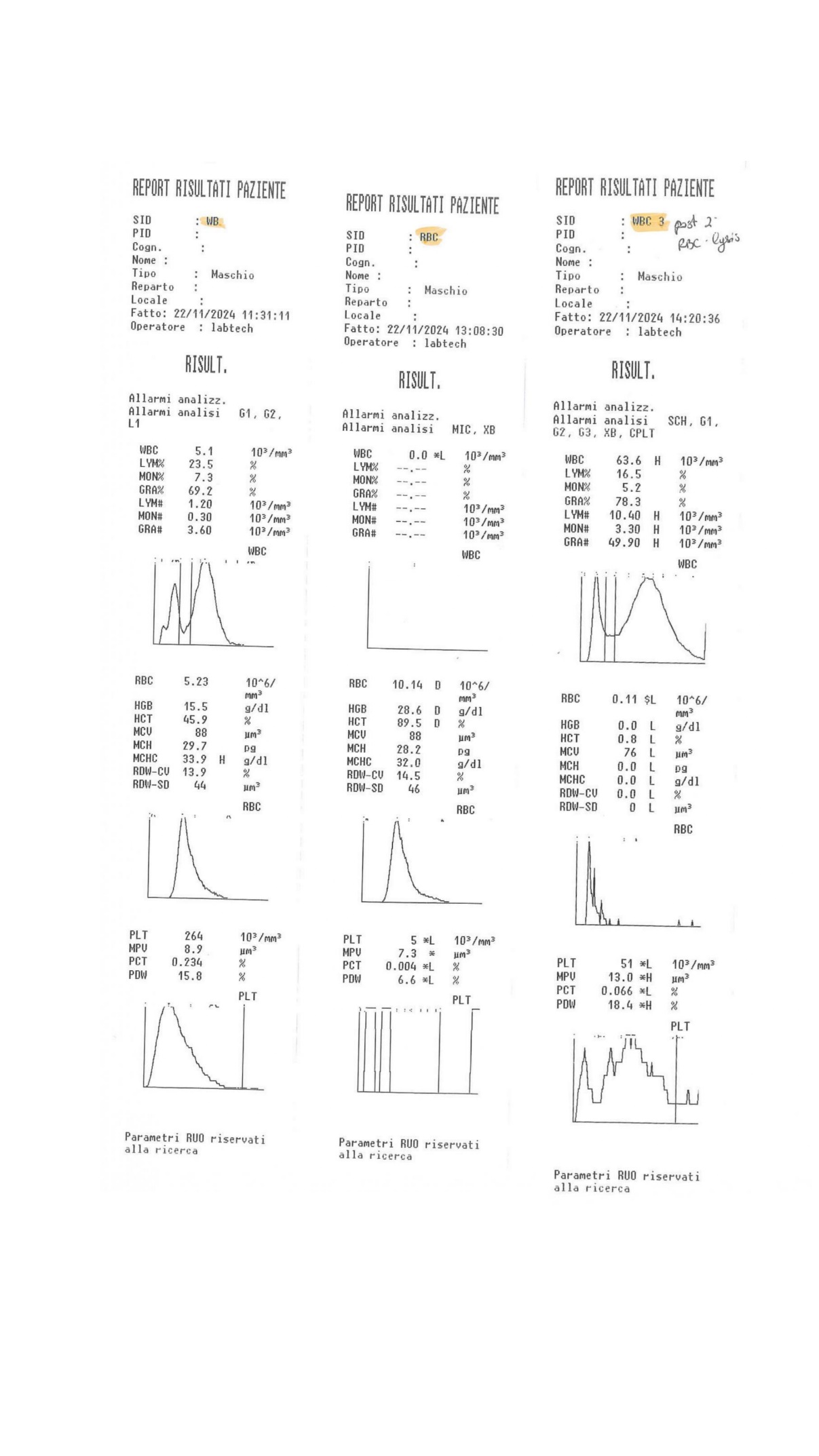


## Table S2 - Small RNA sequences and Assays used for miRNAs relative and absolute quantification by qPCR

The table summarizes IDs, accession numbers and sequences of the investigated miRNAs or short RNAs. The last columns show the Small RNA Assays ID from Thermo Fisher Scientific used in the RT-PCR analyses, both in relative and absolute quantification modes. The sequences reported in the third column were used to design the RNA oligos to set up the standard curves used in absolute quantification mode.

| **miRBase ID** | **miRBase Accession** | **Sequence** | **Thermofisher Assay ID** | **Thermofisher Catalog** |
| --- | --- | --- | --- | --- |
| hsa-miR-106b-5p | MIMAT0000680 | UAAAGUGCUGACAGUGCAGAU | 000442 | 4427975 |
| hsa-miR-196a-5p | MIMAT0000226 | UAGGUAGUUUCAUGUUGUUGGG | 002336 | 4427975 |
| hsa-miR-451a | MIMAT0001631 | AAACCGUUACCAUUACUGAGUU | 001105 | 4427975 |
| U6 snRNA |  | GTGCTCGCTTCGGCAGCACATATACTAAAATTGGAACGATACAGAGAAGATTAGCATGGCCCCTGCGCAAGGATGACACGCAAATTCGTGAAGCGTTCCATATTTT | 001973 | 4427975 |
| hsa-miR-16-5p | MI0000070 | UAGCAGCACGUAAAUAUUGGCG | 000391 | 4427975 |
| hsa-miR-148a-3p | MI0000253 | UCAGUGCACUACAGAACUUUGU | 000470 | 4427975 |
| hsa-miR-29b-3p | MI0000105 | UAGCACCAUUUGAAAUCAGUGUU | 000413 | 4427975 |

## Table S3 – RIP protocol optimization

| **Experiment** | **Non Denaturing Lysis Buffer** | **Anti Ago2 Antibody** | **Control antibody** | **Beads** | **Method** | **Ref.** |
| --- | --- | --- | --- | --- | --- | --- |
| Exp.1 | 50 mM Tris-HCl pH 8.5, 150 mM NaCl, 1% Triton X-100 | mAb R/IgG MA5-14861 Invitrogen | Control Rabbit IgG | Pierce Protein A Magnetic Beads | IP with Ab in solution | [[1](#_ENREF_1)] |
| Exp.2 | 25 mM Tris-HCl pH 7.4, 150 mM NaCl, 1 mM EDTA, 1% NP40, 5% glycerol | mAb R/IgG MA5-14861 Invitrogen | Control Rabbit IgG | Pierce Protein A Magnetic Beads | IP with Ab in solution | [[1](#_ENREF_1)] |
|  | 3 mM Phosphate buffer pH 7.4, 0.5 mM EDTA, 3mM β-MSH |  | Control Rabbit IgG |  | IP with Ab in solution | [[1](#_ENREF_1)] |
| Exp.3 | 3 mM Phosphate buffer pH 7.4, 0.5 mM EDTA, 3mM β-MSH | mAb R/IgG MA5-14861 Invitrogen | Control Rabbit IgG | Pierce Protein A Magnetic Beads | IP with Ab in solution | [[1](#_ENREF_1)] |
| Exp.4 | 3 mM Phosphate buffer pH 7.4, 0.5 mM EDTA, 3mM β-MSH + RNase inhibitor | mAb R/IgG MA5-14861 Invitrogen | Control Rabbit IgG | Pierce Protein A Magnetic Beads | IP with Ab in solution | [[1](#_ENREF_1)] |
| Exp.5 | 3 mM Phosphate buffer pH 7.4, 0.5 mM EDTA, 3mM β-MSH + RNase inhibitor + 1% Formaldehyde | mAb R/IgG MA5-14861 Invitrogen | Control Rabbit IgG | Pierce Protein A Magnetic Beads | IP with Ab in solution | [[1](#_ENREF_1)] |
| Exp.6 | 3 mM Phosphate buffer pH 7.4, 0.5 mM EDTA, 3mM β-MSH + RNase inhibitor + washes in PBS without Tween 20 | mAb R/IgG MA5-14861 Invitrogen | Control Rabbit IgG | Pierce Protein A Magnetic Beads | IP with Ab in solution | [[1](#_ENREF_1)] |
|  |  | pAb R/IgG PA5-117772 Invitrogen | Control Rabbit IgG | Pierce Protein A Magnetic Beads | IP with Ab in solution | [[1](#_ENREF_1)] |
| Exp.7 | 3 mM Phosphate buffer pH 7.4, 0.5 mM EDTA, 3mM β-MSH + RNase inhibitor + washes in PBS without Tween 20 | pAb R/IgG PA5-117772 Invitrogen | Control Rabbit IgG | Pierce Protein A Magnetic Beads | Pre-incubation method | [[2](#_ENREF_2)] |
| Exp.8 | 100 mM HEPES-NaOH oH 7.5, 1 M KCl, 50 mM MgCl2, 5% NP-40, 1 mM DTT, 200U/ml Rnase Out + NT-2 buffer + NET-2 buffer | pAb R/IgG PA5-117772 Invitrogen | Control Rabbit IgG | Pierce Protein A Magnetic Beads | Pre-incubation method | [[3](#_ENREF_3)] |
| Exp.9 | 100 mM HEPES-NaOH oH 7.5, 1 M KCl, 50 mM MgCl2, 5% NP-40, 1 mM DTT, 200U/ml Rnase Out + NT-2 buffer + NET-2 buffer without EDTA | mAb M/IgG1 ab57113 abcam | Control Mouse IgG | Dynabeads protein G Thermofisher | Pre-incubation method | [[3](#_ENREF_3)] |

*pAb, polyclonal antibody; mAb, monoclonal antibody, R/IgG, rabbit IgG; M/IgG, mouse IgG.*

1. Vu, L., Ragupathy, V., Kulkarni, S. & Atreya, C. (2017) Analysis of Argonaute 2-microRNA complexes in ex vivo stored red blood cells, *Transfusion.* **57**, 2995-3000.

2. Panshin, D. D. & Kondratov, K. A. (2020) [The Efficiency of Immunoprecipitation of microRNA/Ago2 Complexes from Human Blood Plasma Is Protocol Dependent], *Molekuliarnaia biologiia.* **54**, 244-251.

3. Gagliardi, M. & Matarazzo, M. R. (2016) RIP: RNA Immunoprecipitation, *Methods in molecular biology.* **1480**, 73-86.

4. Doss, J. F., Corcoran, D. L., Jima, D. D., Telen, M. J., Dave, S. S. & Chi, J. T. (2015) A comprehensive joint analysis of the long and short RNA transcriptomes of human erythrocytes, *BMC genomics.* **16**, 952.

## Table S4 – RBC parameters of the blood donor court

Donor RBC parameters were defined with the means of the automatic haematology analyser ABX Micros (Horiba).

HGB is the amount of haemoglobin expressed as g/dl. The haematocrit (HCT) expresses the ration between the plasma and the corpuscular portion of blood. MCV is the “Mean Corpuscular Volume” which indicates the average volume of the RBCs. MHC is the “Mean Cell Haemoglobin” and refers to the average weight of haemoglobin in the RBCs. MCHC stands for “Mean Cell Haemoglobin Concentration” that is the average concentration of that protein in the RBCs. Reference interval for adults is typically 32 - 36 g/dL. RDW (RBC Distribution Width) is an index for the variability of RBC size in peripheral blood.

All of the reported indexes were perfectly within the physiological range for adults.

|  | **SEX** | **AGE [yrs]** | **RBC [10^6^/µl]** | **HGB [g/dl]** | **HCT [%]** | **MCV [fl]** | **MCH [pg]** | **MCHC [g/dl]** | **RDW [%]** |
| --- | --- | --- | --- | --- | --- | --- | --- | --- | --- |
|  |  |  |  |  |  |  |  |  |  |
| **RBC1** | Male | 37 | 4.78 | 13.2 | 40.3 | 84 | 27.6 | 32.7 | 13.5 |
| **RBC2** | Female | 42 | 3.71 | 14.5 | 36.6 | 99 | 39.3 | 39.7 | 14.6 |
| **RBC3** | Male | 35 | 4.75 | 15.7 | 45.7 | 96 | 33.0 | 34.3 | 13.9 |
| **RBC4** | Female | 41 | 3.85 | 12.6 | 34.9 | 91 | 32.7 | 36.0 | 11.5 |
| **RBC5** | Male | 43 | 4.83 | 14.6 | 43.0 | 89 | 30.2 | 34.0 | 12.7 |
| **RBC6** | Female | 61 | 3.99 | 13.0 | 36.7 | 92 | 32.5 | 35.4 | 13.0 |
| **RBC7** | Female | 38 | 4.48 | 12.1 | 35.4 | 79 | 27.1 | 34.3 | 12.9 |
| **RBC8** | Male | 50 | 4.21 | 14.0 | 40.1 | 95 | 33.2 | 34.9 | 11.9 |
| *Mean* |  | *43* | *4.33* | *13.7* | *39.1* | *91* | *32.0* | *35.2* | *13.0* |
| *St.Dev.* |  | *8* | *0.45* | *1.2* | *3.9* | *7* | *3.8* | *2.1* | *1.0* |

## Figure S1 – miR-29b-3p expression in RBCs and WBCs

The absence of granulocytes from purified RBC samples has been assessed by evaluating miR-29b-3p expression, which is a typical WBC microRNA [[4](#_ENREF_4)]. MiR-29b-3p abundance was quantified using Taqman Small RNA Assay (Applied Biosystems) which includes miRNA reverse transcription step to create a miRNA specific cDNA that has been amplified via a RT-PCR protocol. The results showed in the table below confirmed that we were able to exclude WBC contamination in RBC samples (data ± SEM) (n=8).

5. Doss, J. F., Corcoran, D. L., Jima, D. D., Telen, M. J., Dave, S. S. & Chi, J. T. (2015) A comprehensive joint analysis of the long and short RNA transcriptomes of human erythrocytes, *BMC Genomics.* **16**, 952.

## Figure S2 – Standard curve used for Absolute quantification

Representative standard curve obtained using 5’-phosphorilated miRNA mimics (Merck) resuspended in nuclease-free water at a concentration of 100 pM and stored at -80°C. Standard dilutions were obtained by diluting each oligo to 0.2 µM, and then performing six serial 1:10 dilution in nuclease-free water. Std dil 1-6 were retro transcribed and amplified by the respective small RNA assay (Thermofisher) together with unknown samples. The obtained standard curve was then used to calculate the mean miRNA quantities contained in unknown samples.


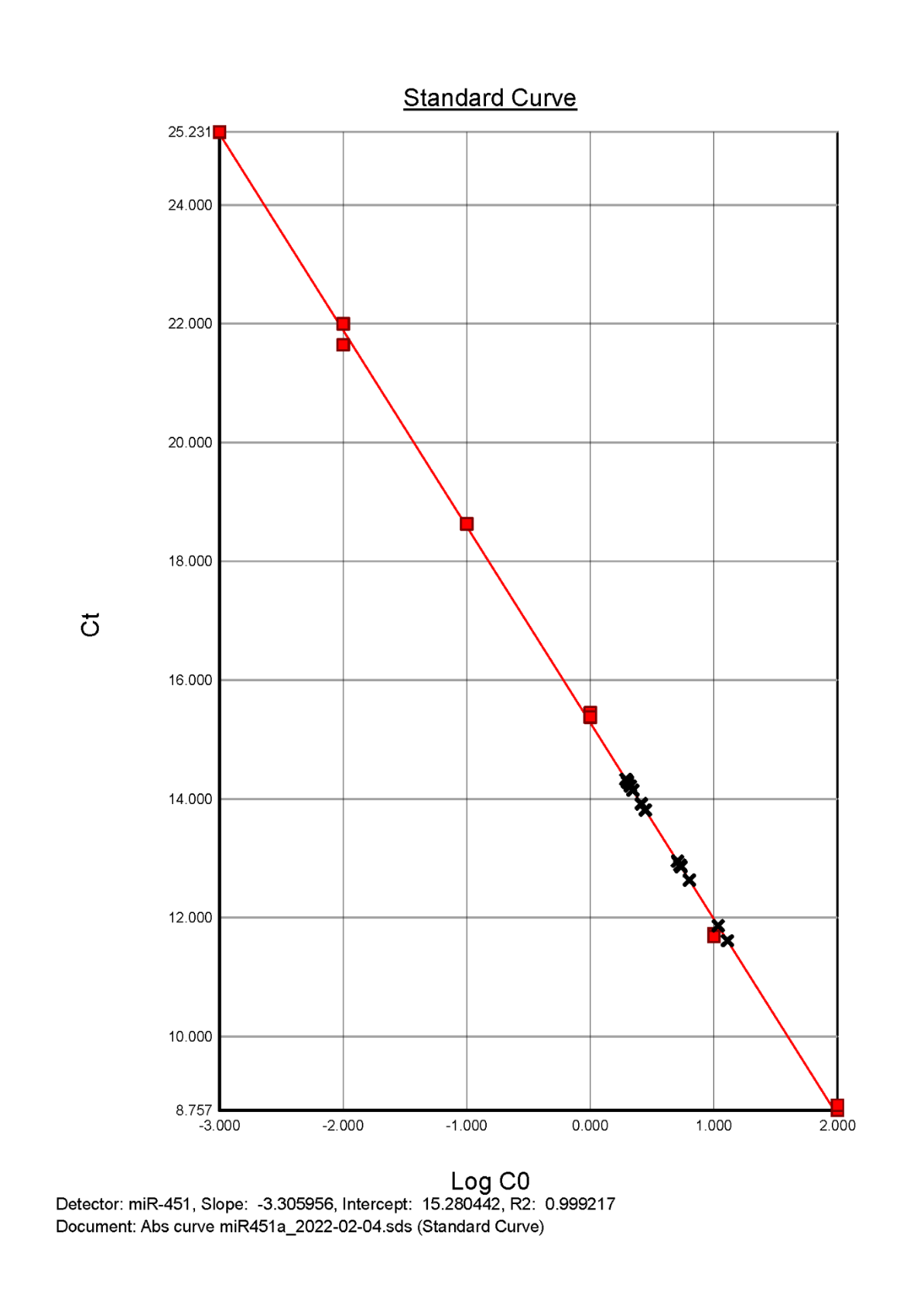


| **Sample Name** | **Detector** | **Task** | **Ct** | **StdDev Ct** | **Qty [fmoles/rection]** | **Mean Qty [fmoles/rection]** | **StdDev Qty [fmoles/rection]** |
| --- | --- | --- | --- | --- | --- | --- | --- |
| Std dil1 | miR-451 | Standard | 8.76 | 0.0621 | 100 |  |  |
| Std dil1 | miR-451 | Standard | 8.84 | 0.0621 | 100 |  |  |
| Std dil2 | miR-451 | Standard | 11.73 | 0.0336 | 10 |  |  |
| Std dil2 | miR-451 | Standard | 11.68 | 0.0336 | 10 |  |  |
| Std dil3 | miR-451 | Standard | 15.45 | 0.0506 | 1 |  |  |
| Std dil3 | miR-451 | Standard | 15.38 | 0.0506 | 1 |  |  |
| Std dil4 | miR-451 | Standard | 18.63 | 6.72E-04 | 0.1 |  |  |
| Std dil4 | miR-451 | Standard | 18.63 | 6.72E-04 | 0.1 |  |  |
| Std dil5 | miR-451 | Standard | 22.00 | 0.25 | 0.01 |  |  |
| Std dil5 | miR-451 | Standard | 21.65 | 0.25 | 0.01 |  |  |
| Std dil6 | miR-451 | Standard | 25.23 | 0.00362 | 0.001 |  |  |
| Std dil6 | miR-451 | Standard | 25.23 | 0.00362 | 0.001 |  |  |
| NT | miR-451 | Unknown | 13.81 | 0.0738 | 2.78115 | 2.68359 | 0.138 |
| NT | miR-451 | Unknown | 13.92 | 0.0738 | 2.58604 | 2.68359 | 0.138 |
| UL t0 | miR-451 | Unknown | 12.63 | 0.227 | 6.32591 | 5.69299 | 0.895 |
| UL t0 | miR-451 | Unknown | 12.95 | 0.227 | 5.06008 | 5.69299 | 0.895 |
| UL t2 | miR-451 | Unknown | 11.61 | 0.177 | 12.856 | 11.8295 | 1.45 |
| UL t2 | miR-451 | Unknown | 11.86 | 0.177 | 10.803 | 11.8295 | 1.45 |
| UL t24 | miR-451 | Unknown | 14.14 | 0.103 | 2.20698 | 2.1001 | 0.151 |
| UL t24 | miR-451 | Unknown | 14.29 | 0.103 | 1.99321 | 2.1001 | 0.151 |
| UL t4 | miR-451 | Unknown | 14.33 | 0.0304 | 1.94284 | 1.9724 | 0.0418 |
| UL t4 | miR-451 | Unknown | 14.28 | 0.0304 | 2.00195 | 1.9724 | 0.0418 |
| UL t48 | miR-451 | Unknown | 12.86 | 0.0168 | 5.40559 | 5.36123 | 0.0627 |
| UL t48 | miR-451 | Unknown | 12.88 | 0.0168 | 5.31687 | 5.36123 | 0.0627 |
| UL t8 | miR-451 | Unknown | 14.21 | 0.00378 | 2.10348 | 2.09958 | 0.00552 |
| UL t8 | miR-451 | Unknown | 14.22 | 0.00378 | 2.09567 | 2.09958 | 0.00552 |
| NTC | miR-451 | NTC | Undetermined |  |  |  |  |
| NTC | miR-451 | NTC | Undetermined |  |  |  |  |
